# FTO Inhibits Insulin Secretion and Promotes NF-κB Activation through Positively Regulating ROS Production in Pancreatic β cells

**DOI:** 10.1371/journal.pone.0127705

**Published:** 2015-05-27

**Authors:** Hong-Qi Fan, Wei He, Kuan-Feng Xu, Zhi-Xiao Wang, Xin-Yu Xu, Heng Chen

**Affiliations:** Department of Endocrinology, the First Affiliated Hospital of Nanjing Medical University, Nanjing, Jiangsu, China; Bascom Palmer Eye Institute, University of Miami School of Medicine;, UNITED STATES

## Abstract

FTO (Fat mass and obesity-associated) is associated with increased risk of obesity and type 2 diabetes incurrence. Pancreas islet β cells dysfunction and insulin resistance are major causes of type 2 diabetes. However, whether FTO plays an important functional role in pancreatic β cells as well as the related molecular mechanism is still unclear. In the present study, the tissue expression profile of FTO was firstly determined using quantitative PCR and western blot. FTO is widely expressed in various tissues and presented with relative high expression in pancreas tissue, especially in endocrine pancreas. FTO overexpression in MIN6 cells achieved by lentivirus delivery significantly inhibits insulin secretion in the presence of glucose stimulus as well as KCl. FTO silence has no effect on insulin secretion of MIN6 cells. However, FTO overexpression doesn’t affect the transcription of insulin gene. Furthermore, reactive oxygen species (ROS) production and NF-κB activation are significantly promoted by FTO overexpression. Inhibition of intracellular ROS production by N-acetyl-L-cysteine (NAC) can alleviate NF-κB activation and restore the insulin secretion mediated by FTO overexpression. A whole transcript-microarray is employed to analyze the differential gene expression mediated by FTO overexpression. The genes which are modulated by FTO are involved in many important biological pathways such as G-protein coupled receptor signaling and NF-κB signaling. Therefore, our study indicates that FTO may contribute to pancreas islet β cells dysfunction and the inhibition of FTO activity is a potential target for the treatment of diabetes.

## Introduction

FTO (Fat mass and obesity-associated) has been identified as an obesity-susceptibility gene, which is strongly associated with increased risk of obesity [1]. FTO was original cloned in mouse and related to the fused toes phenotype resulting from gene deletion on chromosome 8 [2]. The human FTO gene locus is on the chromosome 16q12.2 and is widely expressed in central and peripheral tissues including hypothalamus, adipose and pancreas tissue [1, 3, 4]. FTO gene belongs to dioxygenase superfamily and in vitro study shows that FTO catalyses the demethylation of 3-methylthymine in single strand DNA [5]. Bioinformatic analysis and functional studies have indicated the biological role of FTO in posttranslation modification, gene transcription, cell apoptosis and metabolism process [6]. Three independent studies in the year of 2007 have demonstrated that single nucleotide polymorphisms (SNPs) in the first intron of FTO gene are associated with the increased body mass index (BMI) in Caucasian population [1, 7, 8]. Moreover, different studies in various ethnic populations have indicated that the role of FTO is involved in the obesity, appetite and energy homeostasis [9–12]. A study in FTO-deficient mice has demonstrated that the inactivation of FTO gene protects the mice from obesity and FTO is involved in energy homeostasis by the control of energy expenditure [13, 14].

Obesity is the major risk factor for insulin resistance and β cells dysfunction which lead to the development of type 2 diabetes. Frayling et al. found that FTO gene polymorphism increases the risk of type 2 diabetes incurrence and this association was mediated by BMI [1]. Although this study showed BMI may account for the type 2 diabetes, authors still suggests that FTO gene play a role in the susceptibility to diabetes. Furthermore, various studies showed that FTO gene is independently associated with diabetes after adjusting BMI [15, 16]. Susanne et al. reported that FTO variants are associated with insulin resistance and this association can still be observed after the adjustment of BMI [16]. Recent study showed that FTO gene has a rapid turnover in the pancreatic β cells, involved in the regulation of insulin secretion under glucose stimulation [17]. This study indicated that FTO plays an important role in the biological function regulation of pancreatic β cells. However, the functional role of FTO in pancreatic β cells as well as the related molecular mechanism is still unclear. In this study, we aim to investigate the role of FTO in the pancreatic β cells and explore the related molecular mechanisms.

MIN6 cells were established from insulinoma cells, which retain the response of insulin secretion to glucose and exhibit the similar physiologic characteristics comparable with normal mouse islet cells [18]. MIN6 cells were considered as the useful cell model to study the biological functions and related molecular mechanism underlying the insulin secretion impairment in islet cells. In this study, MIN6 cells were employed to explore FTO function in pancreatic islet cells.

Our study showed that FTO overexpression significantly inhibits the insulin secretion of MIN6 cells but FTO silence doesn’t affect the insulin secretion. The transcriptions of insulin 1 and 2 are not affected by FTO overexpression. Reactive oxygen species (ROS) production and NF-κB activation are significantly promoted by FTO overexpression. Inhibition of intracellular ROS production mediated by FTO overexpression can alleviate activation of NF-κB and restore the insulin secretion. A whole transcript-microarray is employed to analyze the differential gene expression mediated by FTO overexpression. Bioinformatics analysis indicated that FTO may be involved in many important signal pathways.

## Material and Methods

### Animals and cell lines

C57BL/6 mice at 12 weeks of age purchased from the SLAC Laboratory Animal (SLAC, Shanghai, China) were maintained in a specific pathogen free facility with constant temperature of 25°C, light-dark cycle of 12/12 h and free access to water. The experimental protocol was reviewed and approved by the Institutional Animal Care and Use Committee of Nanjing Medical University.

MIN6 cells were a gift from Professor Miyazaki in Kumamoto University Medical School, Japan [19]. MIN6 cells were cultured in DMEM medium with 15% fetal bovine serum, 100 U/ml penicillin, 100 μg/ml streptomycin and 50 μM β-mercaptoethanol in 5% CO_2_ /95% air in a humidified atmosphere at 37°C.

### Tissue collection, RNA extraction and Quantitative PCR

All tissue samples were collected immediately after the animals were sacrificed. The tissues were immediately snap frozen in liquid nitrogen and preserved in -80°C till use. The tissues were further grinded into powder with liquid nitrogen. Total RNA was extracted using Trizol reagent (Invitrogen) following the manufacture’s procedure. A total of 500 ng RNA was reverse transcribed by using FastQuant cDNA Kit (Tiangen, China). Subsequently, quantitative PCR was performed using SYBR Green PCR Master Mix (Applied Biosystems) in an Applied Biosystems 7300 instrument. Expression data were normalized using GAPDH as an internal reference gene and the relative expression levels were evaluated using the delta-delta cycle threshold method (ΔΔCt). Primers for the quantitative PCR are list in the S1 Table.

### Vector construction

To stably express FTO in MIN6 cells, lentivirus plasmid pLVX-IRES-ZsGreen-FTO was constructed. The cDNA sequence of mouse FTO was amplified and subcloned into EcoR I and Xba I sites of pLVX-IRES-ZsGreen vector using the following primers: forward 5’-attGAATTCATGAAGCGCGTCCAGACCG-3’, reverse 5’-ggcTCTAGACTAGGATCTTGCTTCCAGCAGCT-3’. The primers were synthesized by Sangon Biotech (Sangon, Shanghai, China). For shRNA vector construction, pairs of complementary oligonucleotides against FTO (shFTO-1, shFTO-2 and shFTO-3) were synthesized by Sangon, annealed and ligated into lentivirus vector pLentiLox3.7. The targeted sequences of FTO were listed as below: shFTO-1, GTACAGCTATAGCTGCGAA, shFTO-2 GCACCTACAAGTACTTGAA, shFTO-3, CGAGACACCAGGATTAACA.

### Lentivirus package and transduction

Package of pseudotyped recombinant lentivirus was performed by transfection of 293T cells. Briefly, FTO-overexpressed vector pLVX-IRES-ZsGreen-FTO or shRNA expression vector pLentiLox3.7-shFTO-1, pLentiLox3.7-shFTO-2 and pLentiLox3.7-FTO-3 was respectively co-transfected with package vectors pCMV Δ8.91 and pMD.G into 293T cells using Lipofectamine 2000 (Invitrogen, Paisley, Scotland, UK). Lentivirus in the culture media was harvested at 72 h and filtered through a 0.45 μm low protein binding polysulfonic filter (Millipore, Bedford, MA). The virus was frozen and kept in -70°C freezer for future use. For virus infection, MIN6 cells were plated into 6-cm dishes in advance and cultured for nearly 18 h with about 50% confluence before transduction. The lentivirus suspension in the presence of 8 μg/ml polybrene (Chemicon, Temecula, CA) was added to the cells. After transduction for 48 h, green fluorescence was observed to indicate the transduction efficiency.

### Western blot

Cells were lysed and equally loaded to 10% sodium dodecyl sulfate-polyacrylamide gel electrophoresis (SDS-PAGE) and then transferred to nitrocellulose membrane (Axygen, Union City, CA). The membrane was stained with 0.2% Ponceau S Red to ensure equal protein loading. Following block in phosphate-buffered saline containing 5% fat-free milk, the membrane was incubated with primary antibody of FTO (AdipoGen, San Diego, CA) or β-actin (Cell Signaling, Danvers, MA) overnight at 4°C and then incubated with horseradish peroxidase-conjugated anti-rat IgG for 1 h at room temperature. Detection was performed by enhanced chemiluminescence phototope-HRP kit according to the manufacturer’s instruction (Millipore, Bedford, MA).

### Insulin secretion assays

MIN6 cells delivered with pLVX-IRES-ZsGreen-FTO or pLVX-IRES-ZsGreen lentivirus were plated into dishes with the same number and cultured for nearly 18 h with about 60% confluence. Then the cells were washed with Krebs-Ringer bicarbonate (KRB) buffer. After preincubation in the presence of 2 mM glucose for additional 60 min, MIN6 cells were further incubated with KRB buffer containing 2 mM glucose, 20 mM glucose or 50 mM KCl for 10 min or 60 min in 5% CO_2_/95% air in a humidified atmosphere at 37°C. The culture supernatant was collected for analysis of insulin secretion using the Iodine [^125^I] Insulin Radioimmunoassay Kit (Beijing North Institute of Biological Technology, Beijing, China). Insulin analysis was performed according to the manufacturer’s instruction.

### Measurement of intracellular ROS

Intracellular ROS was measured with 2’, 7’-dichlorofluorescein diacetate (DCFH-DA) according to the manufacturer’s instruction (Beyotime, shanghai, China). Briefly, MIN6 cells were harvested and incubated with 10 μM DCFH-DA for 20 min at 37°C in the dark. Then cells were washes with 1×PBS three times and then the relative intensity of fluorescence were quantified by multi-detection microplate reader (488 nm excitation and 525 nm emission).

### Microarray

RNA quantity and quality were measured by NanoDrop ND-1000. RNA integrity was assessed by standard denaturing agarose gel electrophoresis. Sample labeling and array hybridization were performed according to the Agilent One-Color Microarray-Based Gene Expression Analysis protocol (Agilent Technology). Briefly, total RNA from each sample was linearly amplified and labeled with Cy3-UTP. The Labeled cRNAs were purified using RNeasy Mini Kit (Qiagen). The concentration of the labeled cRNAs (pmol Cy3/μg cRNA) was measured by NanoDrop ND-1000. One microgram of each labeled cRNA was fragmented by adding 11 μl 10 × Blocking Agent and 2.2 μl of 25×Fragmentation Buffer, then heated at 60°C for 30 min, and finally 55 μl 2 × GE Hybridization buffer was added to dilute the labeled cRNA. 100 μl of hybridization solution was dispensed into the gasket slide and assembled to the gene expression microarray slide. The slides were incubated for 17 h at 65°C in an Agilent Hybridization Oven. The hybridized arrays were washed, fixed and scanned using the Agilent DNA Microarray Scanner (part number G2505C). Agilent Feature Extraction software (version 11.0.1.1) was used to analyze the acquired array images. Quantile normalization and subsequent data processing were performed with using the GeneSpring GX v11.5 software package (Agilent Technologies). Differentially detected signals were those with P value of <0.05. The dataset has been submitted in Gene Expression Omnibus (GEO) with the accession number of GSE64668.

### GO terms and KEGG pathway annotation of the differentially expressed genes

All the differentially expressed genes were subjected to Gene Ontology (GO) analysis. The GO categories are derived from Gene Ontology (www.geneontology.org). To identify the significant function items, GO enrichment analysis was performed according to the p-values which were calculated by Fisher’s exact test and Chi-square test and then corrected by the false discovery rate (FDR). The less the P-value is, the more significant of the GO Term is (P-value<0.05). Similarly, significant pathway analysis was performed basing on the latest KEGG (Kyoto Encyclopedia of Genes and Genomes) database.

### Statistics

Statistical differences were determined by one-way ANOVA or student’s T-test. Differences are considered as significant when the P value is lower than 0.05.

## Results

### FTO expression in various mouse tissues

**Fig 1:**
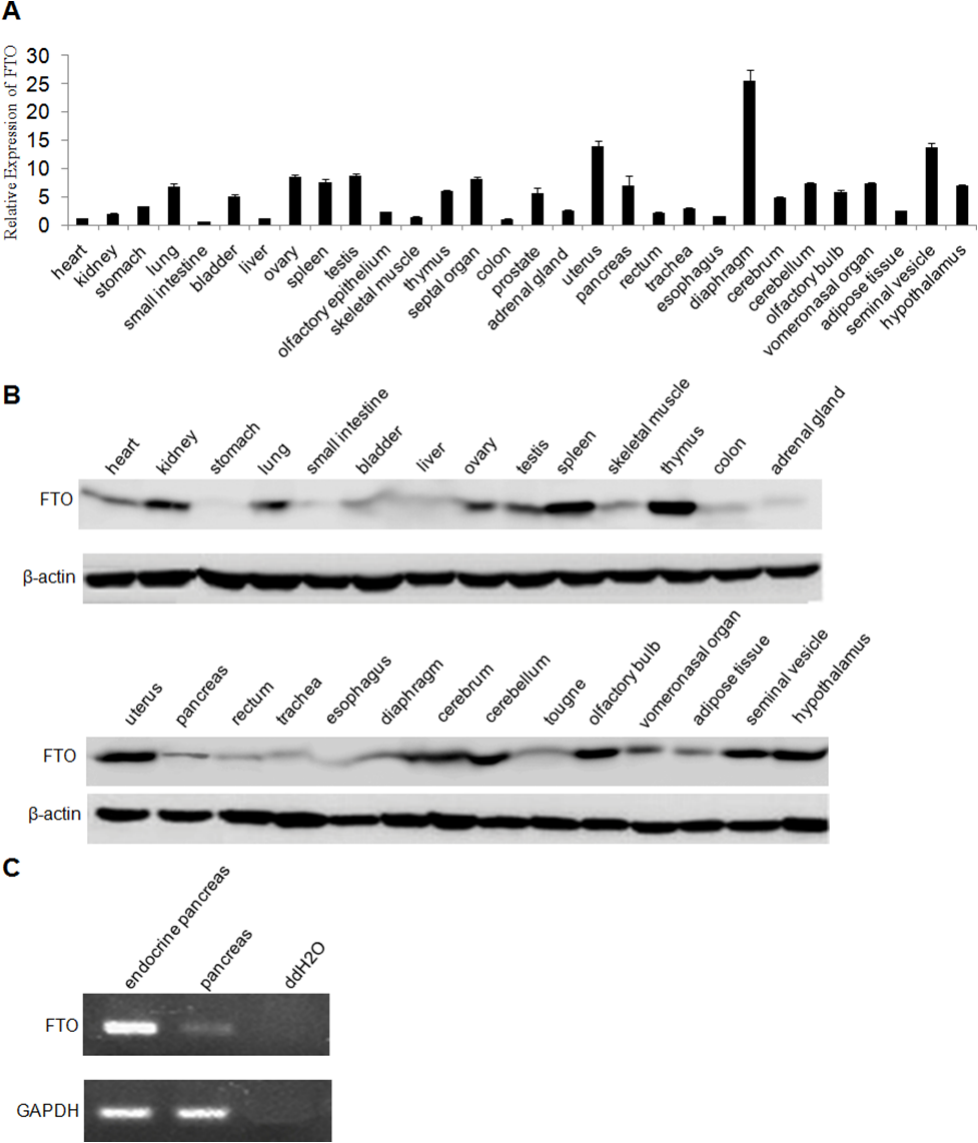
Expression of FTO gene in different tissues of mouse. (A) FTO expression in various mouse tissues measured by Real-time PCR. The data were calculated as relative fold changes between each sample and the one with the lowest expression level of FTO. GAPDH gene is used as internal reference gene for the normalization. Data were presented as mean ± SD. (B) Validation of FTO expression in mouse tissues by Western blot. β-Actin was served as an internal control. (C) Semi-quantitative PCR analysis of FTO expression in endocrine pancreas and pancreas tissue. GAPDH is used as an internal control.

To better understand the FTO biological role, we firstly determine the FTO expression in various mouse tissues using the Quantitative PCR. Our results show that FTO is widely expressed in central and peripheral tissues of mouse. Relative high expression of FTO was observed in lung, ovary, spleen, testis, septal organ, uterus, pancreas, diaphragm, cerebellum, vomeronasal organ, seminal vesicle and hypothalamus (Fig 1A). The same finding is also observed in the tissue FTO expression determined by Western blot (Fig 1B). Our data indicates that the expression of FTO is not tissue specific and FTO may play important biological roles in various tissues such as lung and pancreas. We further analyzed the FTO expression in the endocrine pancreas and pancreas tissue using semi-quantitative PCR. As shown in Fig 1C, a relative higher expression of FTO is observed in endocrine pancreas compared with pancreas gland.

### FTO overexpression inhibits the insulin secretion in MIN6 cells

**Fig 2:**
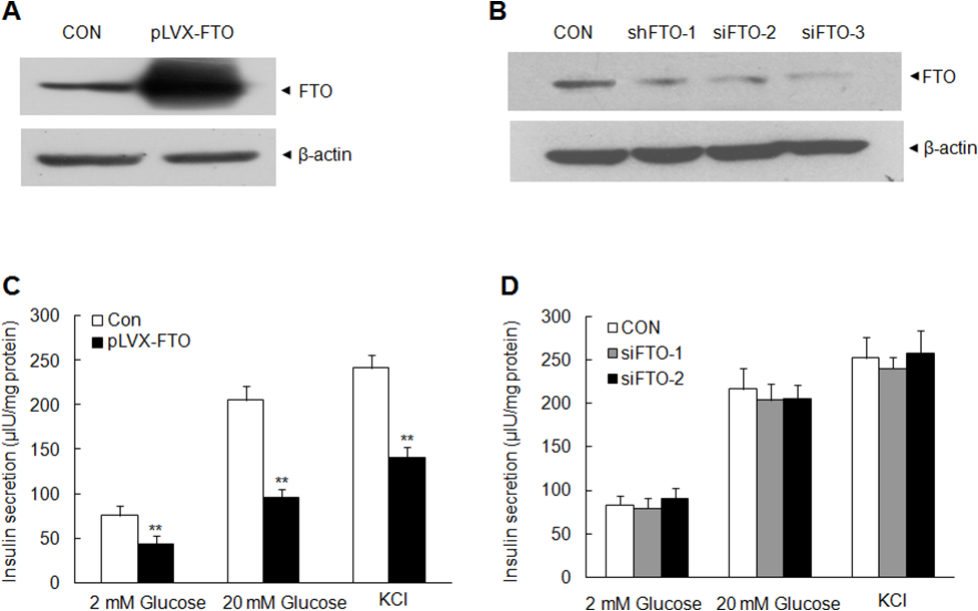
The effect of FTO expression on insulin secretion. (A) FTO overexpression in MIN6 cells via lentivirus delivery using Western blot. β-actin was loaded as an internal control. (B) Detection of FTO in MIN6 cells transfected with FTO shRNA and control shRNA. β-actin was loaded as an internal control. (C) Insulin secretion of MIN6 cells with FTO overexpression at 60 min after 2 mM, 20 mM glucose or 50 mM KCl stimulation. MIN6 cells were stably transfected with pLVX-IRES-ZsGreen (CON) or pLVX-IRES-ZsGreen-FTO (pLVX-FTO). (D) Effect of FTO silence on the insulin secretion in MIN6 cells. MIN6 cells transfected with FTO shRNA 2 or shRNA 3 were analyzed with insulin secretion at 60 min after the glucose or KCl stimulation. Data were presented as mean ± SD. The symbol ** denotes significant statistical difference (p < 0.01).

FTO polymorphisms are associated with type 2 diabetes which is a metabolic disorder in the context of insulin resistance and relative lack of insulin due to dysfunction of pancreas islet cells. Our profile data shows FTO gene may play an important role in pancreas tissues. We further explore the biological role of FTO in pancreas islet cells. FTO overexpression in MIN6 cells was archived by lentivirus delivery and was detected by Western blot analysis (Fig 2A). Furthermore, we knock down FTO in MIN6 cells via RNAi lentivirus. FTO expression was effectively silenced by shFTO-2 and 3 (Fig 2B). Insulin secretion assay was employed to determine whether FTO affects insulin secretion. MIN6 cells were incubated with glucose at different concentration and insulin secretion was determined at 10 min and 60 min when incubation. Our results show that FTO overexpression significantly suppresses the insulin secretion at 10 min (S1A Fig) and the secretion at 60 min (Fig 2C) in the presence of low and high concentration of glucose. Glucose-free secretagogues were also performed and the same effect was observed in potassium chloride (KCl) treatment (S1A Fig and Fig 2C). Insulin secretion experiment shows that FTO knockdown only slightly increase the insulin secretion compared with the control group (Fig 2D and S1B Fig). Our results indicated that FTO affect the insulin secretion in pancreas islet cells and this may contribute type 2 diabetes occurrences.

### FTO doesn’t affect the transcription of insulin1/2

**Fig 3:**
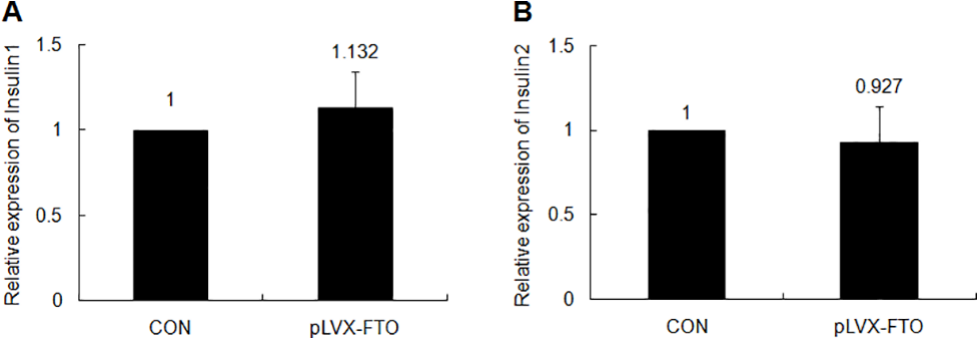
FTO doesn’t affect the transcription of insulin 1/2. The expression level of insulin 1 (A) and insulin 2 (B) in MIN6 cells delivered with FTO expression lentivirus or control vector using Real-time PCR.

To investigate the mechanism that FTO regulates the insulin secretion, we determined the mRNA level of insulin 1 and insulin 2 in MIN6 cells stably overexpressed pLVX-IRES-ZsGreen-FTO or pLVX-IRES-ZsGreen using QPCR. Both the expression of insulin 1 and 2 are not affected by FTO overexpression compared with that in control group (Fig 3A and 3B).

### FTO promotes the ROS production and NF-κB activation in MIN6 cells

**Fig 4:**
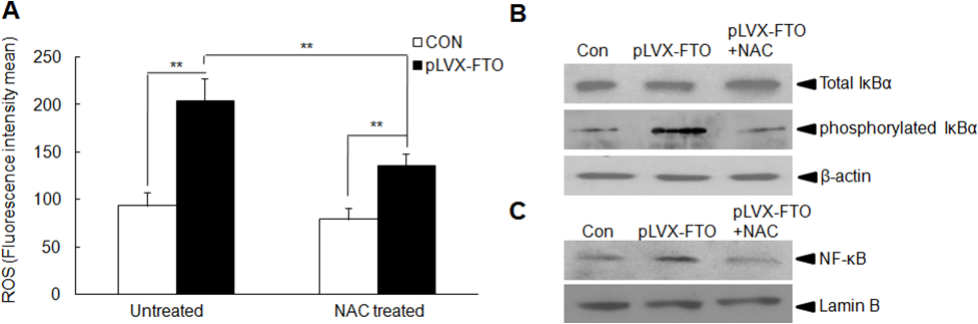
FTO promotes the ROS production and then induces NF-κB activation in MIN6 cells. (A) Detection of ROS production in FTO-overexpressed cells or control cells with or without NAC treatment. (B) Total and phosphorylated IκBα immunoblotting in whole cell lysates. β-actin was used as an internal control. (C) Detection of p65 in nuclear fraction using Western blot. Lamin B was used as an internal control. Data were presented as mean ± SD. The symbol ** denotes significantly statistical difference (p < 0.01).

It has been reported that increased FTO expression was detected in muscle from type 2 diabetic patients and FTO overexpression enhances the oxidative stress in human myotubes [20]. To gain more insight of the biological role of FTO in pancreas islet cells, we therefore investigate whether FTO overexpression could alter the ROS production in MIN6 cells. As shown in Fig 4A, a significant increased production of ROS was observed in MIN6 cells with FTO overexpression compared with that in the control group. Increased ROS production may lead to the activation of NF-κB pathway involved in the pancreas inflammation response [21]. We perform the Western blot assay to determine the activation of NF-κB pathway. There is a remarkably phosphorylation of IκBα in the whole cell lysates of MIN6 cells delivered with pLVX-IRES-ZsGreen-FTO lentivirus, which indicated the activation of NF-κB pathway (Fig 4B). Furthermore, the nuclear translocation of p65 was also observed in FTO-overexpressed MIN6 cells, which demonstrates the activation of NF-κB pathway (Fig 4C). To further verify the role of ROS in the activation of NF-κB pathway, N-acetyl-L-cysteine (NAC), ROS scavenger, was used to decrease the intracellular ROS level in MIN6 cells with FTO overexprssion. NAC significantly decreases the ROS level in FTO-overexpressed MIN6 cells (Fig 4A) and alleviates the NF-κB pathway activation (Fig 4B and 4C). Our data indicates that FTO overexpression enhanced the intracellular ROS production which further mediated the activation of NF-κB pathway.

### NAC treatment partly rescues the effect of FTO overexpression on the inhibition of insulin secretion

**Fig 5:**
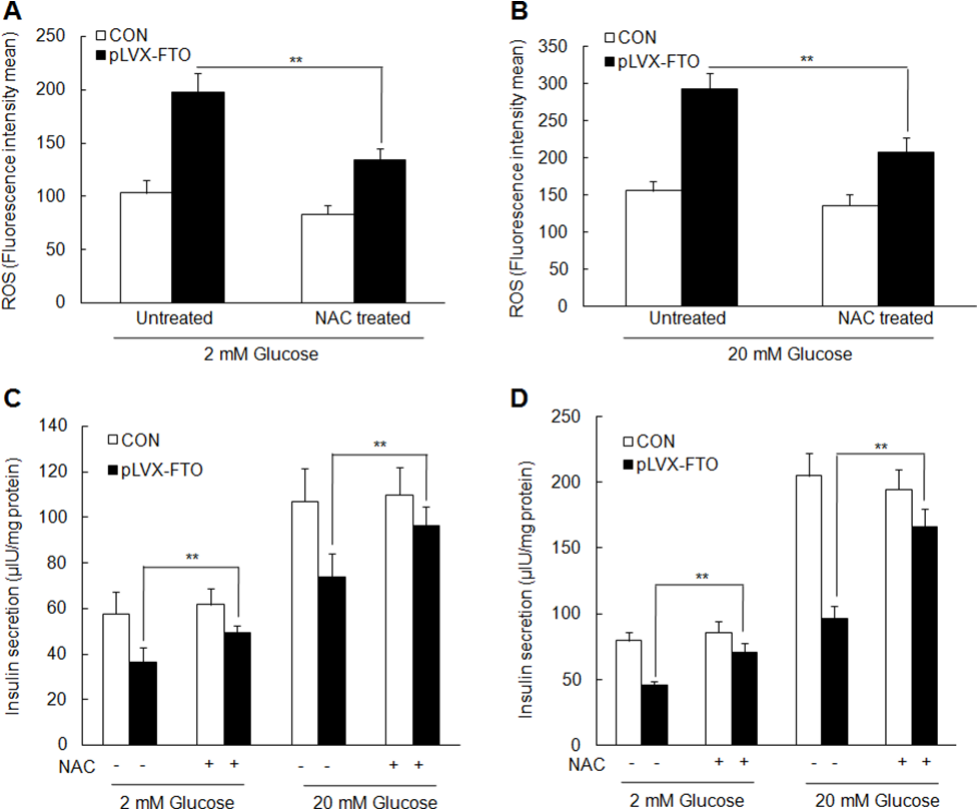
NAC treatment partially rescues the effect of FTO overexpression on the inhibiton of insulin secretion. Detection of ROS production in MIN6 with overexpressed-FTO or empty vector with NAC treatment or not in the presence of 2 mM glucose (A) or 20 mM glucose (B)**.** Detection of insulin secretion with NAC treatment or not at 10 min (C) or 60 min (D) after the stimulation of 2 mM or 20 mM glucose. Data were presented as mean ± SD. The symbol* and ** denotes statistical difference p < 0.05 and p < 0.01, respectively.

As mentioned before, FTO overexpression decreased the insulin secretion and promotes ROS production and activation of NF-κB pathway. To further explore the relation between the ROS production and insulin secretion inhibition resulted from FTO overexpression, MIN6 cells with FTO overexpression or control were pretreated with NAC. Then the intracellular ROS and insulin secretion in respond to glucose and KCl stimulation were analyzed. Consistently, higher ROS level was observed in MIN6 cells with FTO overexpression than that in control MIN6 cells in the presence of 2 mM and20 mM glucose. NAC treatment significantly decreases the ROS production in MIN6 cells (Fig 5A and 5B). Insulin secretion showed that NAC treatment partly restores the insulin secretion in FTO-overexpressed MIN6 cells in respond to 2 mM and 20 mM glucose stimulation at 10 min (Fig 5C) and at 60 min (Fig 5D). Similarly, NAC treatment also partly restores the insulin secretion in MIN6 cells with FTO overexpression in respond to KCl stimulation (S2A Fig and S2B Fig). Our data indicates that the effect of FTO on insulin secretion is mediated by ROS production.

### Analysis of gene expression using Whole Mouse Genome Oligo Microarrays

To explore an overall view of early molecular events occurring after FTO overexpression, a transcriptomic study was performed using a whole-transcript microarray. There are a total of 885 named genes modulated by the overexression of FTO according to the criteria that the relative fold change of expression is above 2 or below 0.5. Among these differential expression genes, there are 343 upregulated genes and 542 downregulated genes. Especially, 21 differential expression genes with more than 10-fold changes are listed in S2 Table and S3 Table.

### GO and KEGG pathway

**Fig 6:**
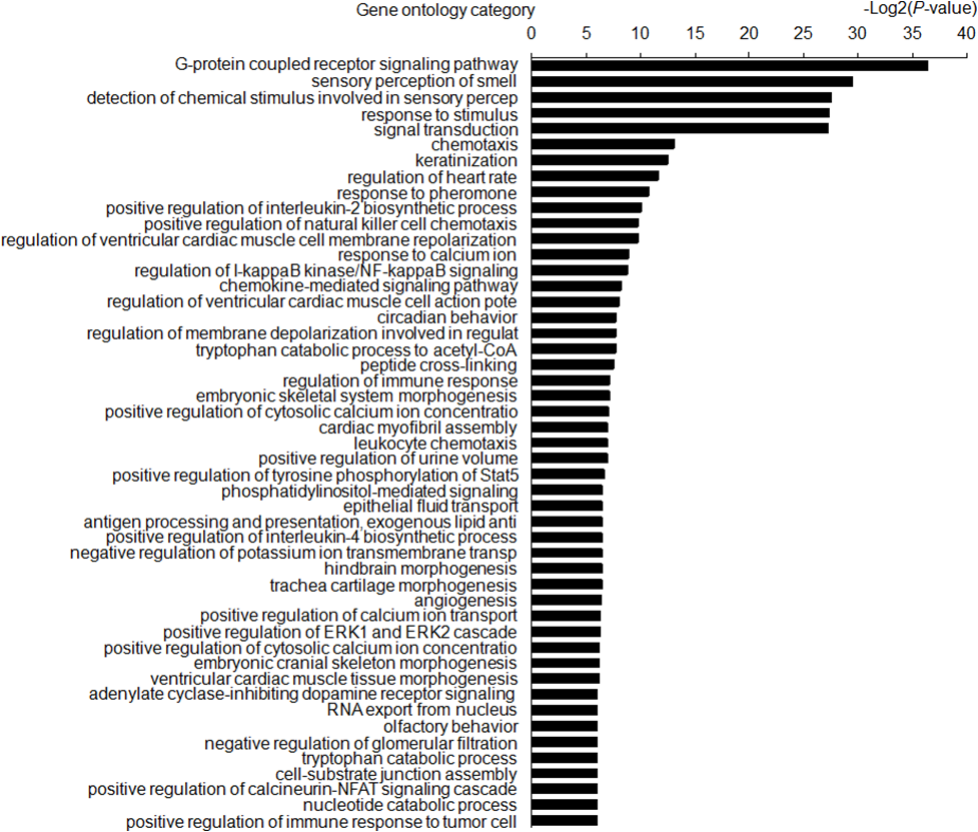
GO annotation of differential genes modulated by FTO overexpression in MIN6 cells. GO categories (p<1.0×10^–5^) were ranked according to their *p* values and shown in the left. The data in the horizontal axis mean the minus logarithm based on 10 and they represent the significant degrees of annotated functions of differential genes.

**Fig 7:**
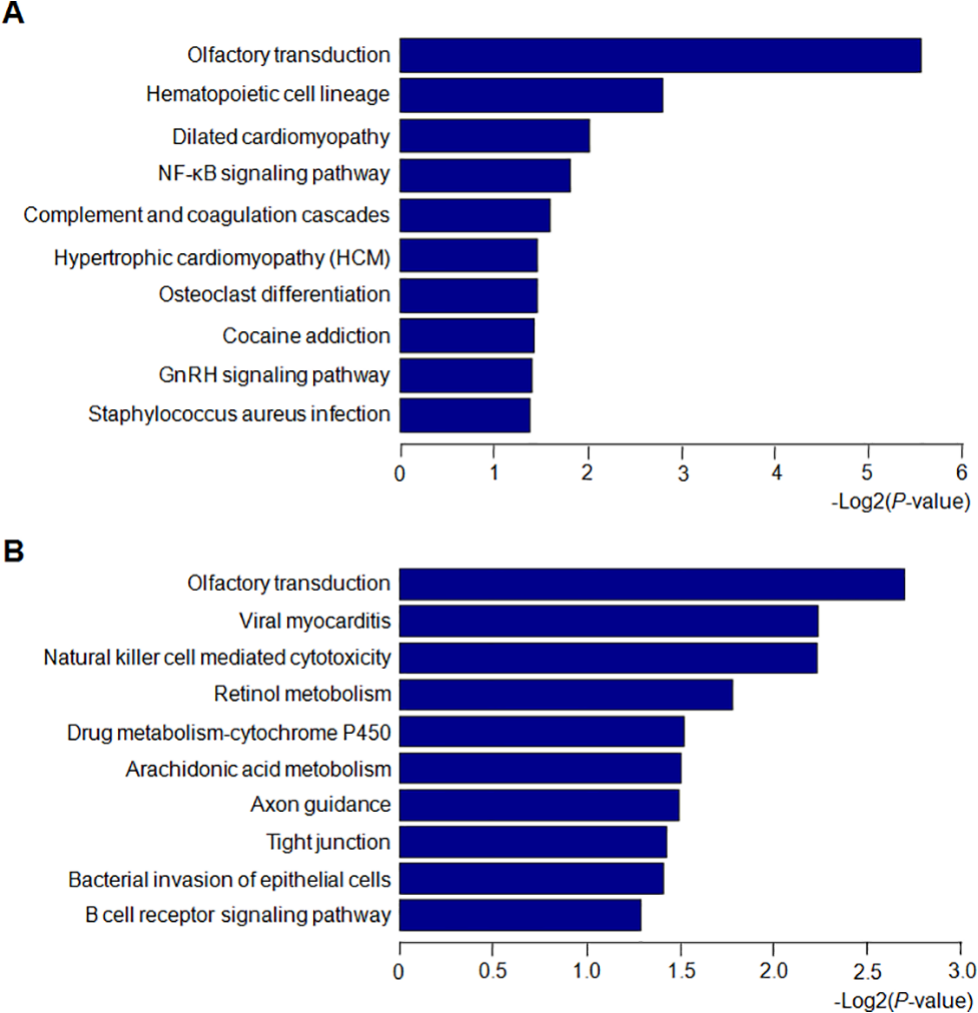
Pathway analysis of differential genes modulated by FTO overexpression in MIN6 cells. (A) Pathway analysis of upregulated genes modulated by FTO overexpression. (B) Pathway analysis of downregulated genes modulated by FTO overexpression. The data in the horizontal axis mean the minus logarithm based on 10 and they represent the significant degrees of annotated pathways of differential genes.

In order to reveal the related functions and pathways, the differential genes modulated by FTO overexpression in MIN6 cells were classified by a functional enrichment analysis using GO and KEGG annotation databases. Under the criteria of p-value less than 1.0×10^–5^,58 GO categories were chosen and schematically represented according to their p-values (Fig 6). The most significant GO categories were related to G-protein coupled receptor signaling, sensory perception of smell, response to stimulus and signal transduction. Besides, the GO term related to regulation of IκB kinase / NF-κB signaling is also of significance and consistent with our above findings. The KEGG enrichment analysis indicated modulated pathways involved in olfactory transduction, hematopoietic cell lineage, dilated cardiomyopathy, and NF-κB signaling pathway for upregualted genes, as well as the pathways related to olfactory transduction, viral myocarditis and nature killer cell mediated cytotoxicity for the down regulated genes (Fig 7).

### Analysis of the gene expression affected by FTO overexpression

**Fig 8:**
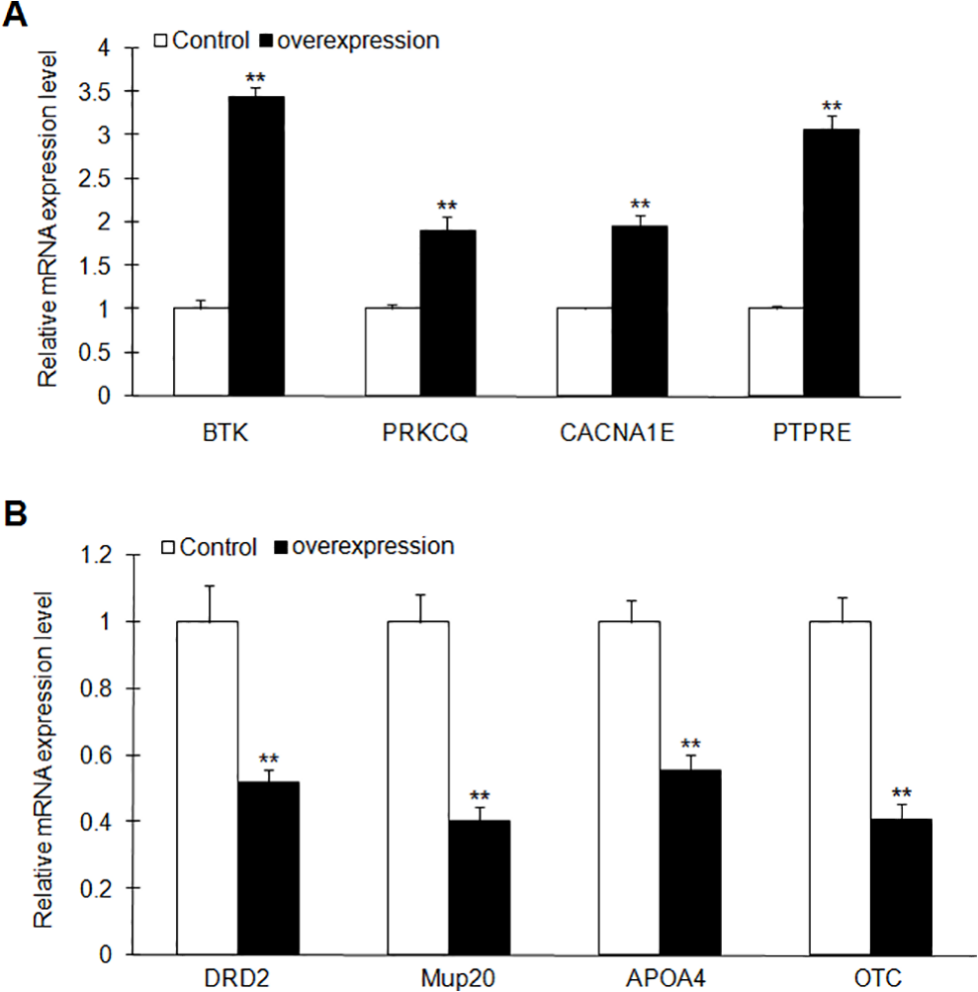
Gene expression analysis by Real-time PCR. The expression of genes modulated by FTO overexpression was verified using qPCR. (A) Expression analysis of the upregulated genes in the microarray. (B) Expression analysis of the downregulated genes in the microarray. These genes are related to NF-κB signaling, metabolism and insulin secretion regulation. Data indicated mean ± SD. The symbol ** denotes statistical difference (p < 0.01).

In order to validate the microarray data, quantitative PCR was performed from MIN6 cells delivered with pLVX-IRES-ZsGreen-FTO or pLVX-IRES-ZsGreen lentivirus. We are focused on the genes related with NF-κB pathway, metabolism and energy balance as well as insulin secretion. These genes modulated by FTO overexpression according to microarray data were confirmed by quantitative PCR. Among the FTO-upregualted genes, Btk and PRKCQ were involved in the phosphorylation and activation of NF-κB pathway. Btk induces the phosphorylation of NF-κB p65 which can be induced by the expression of inflammation cytokines [22]. PRKCQ belongs to Protein kinase C family which is required for the activation of NF-κB pathway. Upregulated gene Cacna1e is involved in the regulation of second-phase insulin release and the polymorphism of Cacna1e is associated with type 2 diabates [23–25]. The expression pattern of these upregulated genes were confirmed by the QPCR analysis using the samples from the MIN6 cells with FTO overexpression (Fig 8A). For FTO-downregualted genes, several genes were previous implicated in metabolism regulation and energy balance, including Mup20, Apoa4 and Otc. Dopamine receptor D2 (Drd2), another FTO-downregualted gene, has been reported that its expression is reduced in the diet-induced obese companied with insulin resistance [26]. Modulation of Drd2 activity can affect insulin secretion and metabolism process [26, 27]. These down-regulate genes were validated by QPCR (Fig 8B).

## Discussion

Previous studies have demonstrated that FTO polymorphisms are associated with obesity and the risk of type 2 diabetes in various human populations [1, 7, 8]. The expression of FTO gene is observed in various tissues including the brain, lung and pancreatic tissues. Our study also detected the FTO expression in mouse various tissues and the finding confirmed the wide expression pattern of FTO. It has been reported that the expression of FTO in brain tissue controls appetite and feeding behavior which is consistent with the role of this gene in the regulation of body mass. But the role of FTO in other tissues including pancreas is still not clear. Our tissue expression profile of FTO indicated that FTO may play an important role in pancreas in consideration of its relative high expression level.

To address the functional role of FTO in pancreatic islet β cells, we increase or silence the expression of FTO in mouse MIN6 cells and explore related molecular mechanism underlying the functional changes mediated by FTO. GSIS experiments showed that FTO overexpresssion significantly inhibits insulin secretion in response to glucose stimulus. However, FTO silence doesn’t affect the insulin secretion. We further analyze the mRNA level of insulin 1 and 2 in MIN6 cells with stably overexpressed FTO. FTO overexpression has no effect on insulin 1 and 2 transcription according to QPCR data. Furthermore, Mark et al. reported that induced-expression of FTO enhance the first phase of insulin secretion in response to glucose stimulus in islet cells, while the second phase of insulin secretion was not changed [17]. Taken together, these results offer the evidences that FTO overexpression inhibits insulin release from islet cells but without any effect on the transcription of insulin. However, our data show that FTO silence doesn’t affect insulin secretion. This result could be explained with the reasons that FTO silence just partly knock down FTO expression but the remaining FTO still can maintain the biological function in islet cells. This explanation need to be further verified with experiments.

Bravard et al. reported that increased expression of FTO in muscle of type 2 diabetic patients enhanced oxidative stress and reduced mitochondrial oxidative function [20]. Increased generation of reactive oxygen species (ROS) activates NF-κB which is involved in inflammation response and endoplasmic reticulum stress in pancreatic cells [28, 29]. Salem et al. reported that the activation of NF-κB pathway trigger the immune-mediated injury of pancreatic islet β cells and diabetes incurrence [30]. In our study, ROS production was analyzed in MIN6 cells with FTO overexpression or control. Our data showed that FTO overexpression significantly increases the ROS generation. Meanwhile, the activation of NF-κB is observed in MIN6 cells with FTO overexpression. To further explore the role of ROS in the activation of NF-κB pathway, NAC was employed to decrease the intracellular ROS level in MIN6 cells with FTO overexprssion. NAC treatment decreased the intracellular ROS production and alleviates the activation of NF-κB. Our study indicated that FTO overexpression activates NF-κB via ROS generation. The activation of NF-κB may further cause the endoplasmic reticulum stress and damaged mitochondrial oxidative function which resulted in the GSIS reduction.

GO analysis basing on the differential gene expression profile showed that FTO-modulated genes are involved in the G-protein coupled receptor signaling, sensory perception of smell, response to stimulus and signal transduction as well as IκB kinase / NF-κB signaling. Our gene expression profile showed that several olfactory-related genes are significantly modulated by FTO expression. Why these genes changed in pancreatic cells and the related biological function are rarely reported. As illustrated in GO annotation, there are a total of 46 differential genes related to metabolism, energy balance and insulin secretion regulation. CACNA1E, one of the upregulated genes, encodes the voltage-dependent Ca(2+) channel Ca(V) 2.3. It has been reported this gene is involved in the regulation of second-phase insulin secretion and its polymorphism contributes to an increased risk of the development of type 2 diabetes [23–25]. Another upregulated gene, Ptpre, encoding protein tyrosine phosphatases, is a negative regulator of insulin receptor (IR) signaling and involved in the insulin-induced glucose metabolism through inactivation of IR [31, 32]. Dopamine receptor D2 (Drd2) gene is downregulated in MIN6 cells with FTO overexpression. It has been reported that diet-induced obese mice are insulin resistance and have a reduced expression of Drd2. The study further showed that the Drd2 agonist reduced the body weight and reinforced insulin action in the diet-induced mice. Conversely, treatment with Drd2 antagonist in diet resistant mice reduced the voluntary activity and induced insulin resistance in these mice [26]. Drd2 is involved in the regulation of insulin secretion and the balance of metabolism [27]. Whether the expression of these modulated genes is directly regulated by FTO and their biological roles of these genes in pancreatic islet β cells need to be further explored.

In conclusion, our study revealed that FTO expression doesn’t show temporal specificity, but relative high expression level of FTO is observed in pancreas tissues. Furthermore, it has been demonstrated that FTO overexpression can inhibit insulin secretion in response to glucose stimulus rather than insulin synthesis in transcription level in pancreas islet β cells. FTO overexpression enhances ROS production and activates NF-κB pathway. Whole-transcript microarray analysis was performed and the differential expression genes modulated by FTO are involved in many important biological pathways such as G-protein coupled receptor signaling and NF-κB signaling. Therefore, our study proposes that upregulated FTO may contribute to pancreas islet β cells dysfunction and diabetes incurrence.

## Supporting Information

S1 Fig FTO overexpression inhibits insulin secretion. (A) Insulin secretion of MIN6 cells with FTO overexpression at 10 min after the stimulation of 2 mM or 20 mM glucose or 50 mM KCl. (B) Detection of secreted insulin in MIN6 cells transfected with FTO shRNA 2 or shRNA 3 at 10 min after the stimulation of 2 mM or 20 mM glucose or 50 mM KCl. Data were presented as mean ± SD. The symbol * denotes statistical difference (p < 0.05).
(TIF)

S2 Fig Inhibition of insulin secretion mediated by FTO overexpression was partially reversed by NAC treatment. Detection of insulin secretion in MIN6 cells pretreated with NAC or not at 10 min (A) and 60 min (B) after the stimulation of 50 mM KCl. Data was presented as mean ± SD. The symbol ** denotes significantly statistical difference (p < 0.01).
(TIF)

S1 Table Primers for Real-time PCR. (DOC)

S2 Table Up-regulated genes with fold changes of more than 10 times. (DOCX)

S3 Table Down-regulated genes with fold changes of more than 10 times. (DOCX)
