# Supplementary figures and images for: FTO Inhibits Insulin Secretion and Promotes NF-κB Activation through Positively Regulating ROS Production in Pancreatic β cells
Source: PLoS One. 2015 May 27;10(5):e0127705. doi: 10.1371/journal.pone.0127705 (PMC4446323; doi:10.1371/journal.pone.0127705)

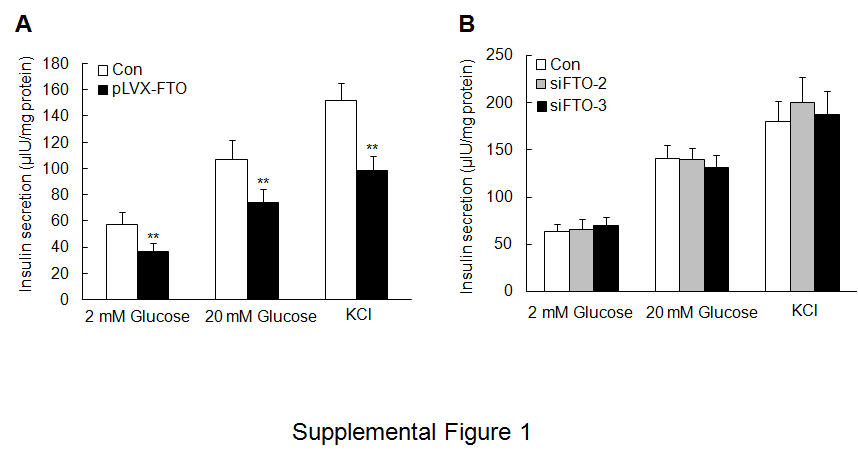

Supplement: S1 Fig — (A) Insulin secretion of MIN6 cells with FTO overexpression at 10 min after the stimulation of 2 mM or 20 mM glucose or 50 mM KCl. (B) Detection of secreted insulin in MIN6 cells transfected with FTO shRNA 2 or shRNA 3 at 10 min after the stimulation of 2 mM or 20 mM glucose or 50 mM KCl. Data were presented as mean ± SD. The symbol * denotes statistical difference (p < 0.05). (TIF) [file pone.0127705.s001.tif]

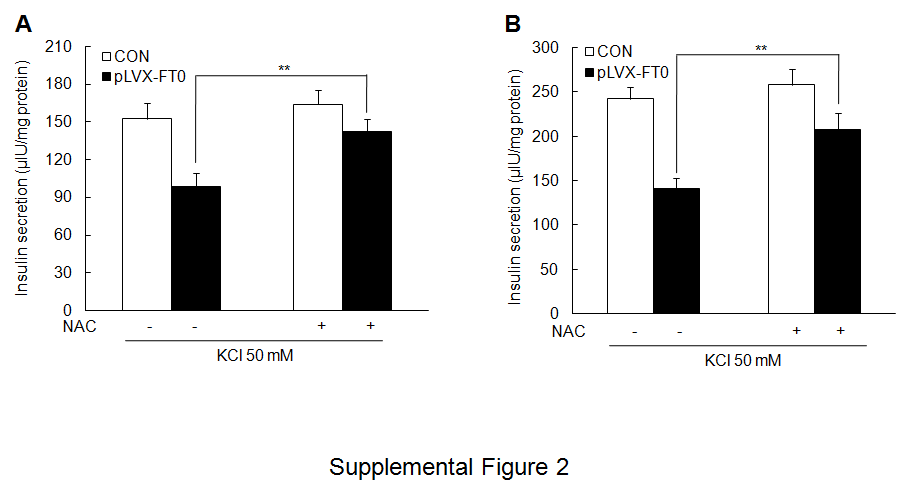

Supplement: S2 Fig — Detection of insulin secretion in MIN6 cells pretreated with NAC or not at 10 min (A) and 60 min (B) after the stimulation of 50 mM KCl. Data was presented as mean ± SD. The symbol ** denotes significantly statistical difference (p < 0.01). (TIF) [file pone.0127705.s002.tif]
