# Supplementary material for: FTO Inhibits Insulin Secretion and Promotes NF-κB Activation through Positively Regulating ROS Production in Pancreatic β cells
Source: PLoS One. 2015 May 27;10(5):e0127705. doi: 10.1371/journal.pone.0127705 (PMC4446323; doi:10.1371/journal.pone.0127705)
Supplement: S1 Table — (DOC) [file pone.0127705.s003.doc]

**S1 Table.** Primers for Real-time PCR

| Name | Sequence (5’-3’) | Tm (°C) | Amplicon (bp) |
| --- | --- | --- | --- |
| mFTO-F | CTGCGAAGGCTCTGAGGATGA | 62.7 | 185 |
| mFTO-R | AAACACAGTGCTGGTGGGTGG | 62.8 |
| mBTK-F | GGTCGTGGCCCTTTATGATTA | 59.4 | 135 |
| mBTK-R | TAGCCTTCCTGCCCATTTTTA | 59.6 |
| mPRKCQ-F | ATGGACAACCCCTTCTACCC | 57.7 | 104 |
| mPRKCQ-R | TCTCCTCTCACTCCCAGCCT | 58.4 |
| mCacna1e-F | GTGCTTTCCGGGGAATTTG | 60.3 | 117 |
| mCacna1e-R | TATCCAGGCACGGTAGCCG | 61.7 |
| mPtpre-F | CTGGGAGCAAAGGTCAGCC | 60.3 | 150 |
| mPtpre-R | TAATCCACCAGGACCACGCA | 61.8 |
| mDrd2-F | ATGCTGCTCACCCTCCTCAT | 59.2 | 88 |
| mDrd2-R | TGGTCTGCAAAGCCTTCTCTC | 59.5 |
| mMup20-F | TTTGTCGAGTACATCCATGTCTTG | 59.6 | 119 |
| mMup20-R | TATTCACCAGCCTTTTCTGTTTTG | 60.8 |
| mApoa4-F | GGGAGAGATGTTCAACAAGGCT | 59.9 | 145 |
| mApoa4-R | CAGGGTGCTCATAAAGGAGTTG | 59.2 |
| mOtc-F | GGCTGCGTCTGACTGGACAT | 60.5 | 119 |
| mOtc-R | TCCACTTTCTATTCTCTGCCTCTG | 59.9 |
| mInsulin-1-F | CACTTCCTACCCCTGCTGG | 60.7 | 81 |
| mInsulin-1-R | ACCACAAAGATGCTGTTTGACA | 60.6 |
| mInsulin-2-F | GCTTCTTCTACACACCCATGTC | 58.7 | 147 |
| mInsulin-2-R | AGCACTGATCTACAATGCCAC | 57.8 |
