# Supplementary material for: FTO Inhibits Insulin Secretion and Promotes NF-κB Activation through Positively Regulating ROS Production in Pancreatic β cells
Source: PLoS One. 2015 May 27;10(5):e0127705. doi: 10.1371/journal.pone.0127705 (PMC4446323; doi:10.1371/journal.pone.0127705)
Supplement: S2 Table — (DOCX) [file pone.0127705.s004.docx]

**S2 Table.** **Up-regulated genes with fold changes of more than 10 times**

| **GeneSymbol** | **Fold change** | **[FTO]vs[LVX]** | **Gene Description** |
| --- | --- | --- | --- |
| Nlrp9b | 39.31 | up | NLR family, pyrin domain containing 9B (Nlrp9b) |
| Upp2 | 24.76 | up | uridine phosphorylase 2 (Upp2) |
| Npat | 21.44 | up | nuclear protein in the AT region (Npat) |
| Six3 | 20.24 | up | sine oculis-related homeobox 3 homolog |
| Gm10388 | 19.78 | up | PREDICTED: Mus musculus predicted gene 10388 (Gm10388) |
| Gm4668 | 18.35 | up | Mus musculus 3 days neonate thymus cDNA, product: unclassifiable |
| Il1a | 15.80 | up | Mus musculus interleukin 1 alpha (Il1a) |
| Tomt | 14.68 | up | transmembrane O-methyltransferase (Tomt) |
| Gm15417 | 13.89 | up | AGENCOURT_10817022 NIH_MGC_156 Mus musculus cDNA |
| 2310001H17Rik | 12.01 | up | Mus musculus adult male bone cDNA, product: unclassifiable |
| A930027P06Rik | 11.92 | up | Mus musculus adult retina cDNA, product:unclassifiable |
| Lcn4 | 11.28 | up | lipocalin 4 (Lcn4) |
| AW552393 | 11.15 | up | UI-M-AO1-aen-e-09-0-UI.s1 NIH_BMAP_MPG_N Mus musculus cDNA clone UI-M-AO1-aen-e-09-0-UI 3 |
| Gm10739 | 10.37 | up | Mus musculus adult male hypothalamus cDNA, product: hypothetical protein |
