# Supplementary material for: FTO Inhibits Insulin Secretion and Promotes NF-κB Activation through Positively Regulating ROS Production in Pancreatic β cells
Source: PLoS One. 2015 May 27;10(5):e0127705. doi: 10.1371/journal.pone.0127705 (PMC4446323; doi:10.1371/journal.pone.0127705)
Supplement: S3 Table — (DOCX) [file pone.0127705.s005.docx]

**S3 Table.** **Down-regulated genes with fold changes of more than 10 times**

| **GeneSymbol** | **Fold change** | **[FTO]vs[LVX]** | **Gene Description** |
| --- | --- | --- | --- |
| 4930529F21Rik | 14.049982 | down | Mus musculus adult male testis cDNA, product: unclassifiable |
| Olfr616 | 12.714516 | down | olfactory receptor 616 (Olfr616) |
| Vmn1r16 | 11.590572 | down | vomeronasal 1 receptor 16 (Vmn1r16) |
| Gm2984 | 11.523697 | down | Mus musculus 16 days embryo head cDNA, product: unclassifiable |
| Rassf9 | 11.505781 | down | Ras association (RalGDS/AF-6) domain family (N-terminal) member 9 (Rassf9) |
| Sval2 | 11.304433 | down | Mus musculus seminal vesicle antigen-like 2 (Sval2) |
| Vgll1 | 10.62 | down | Mus musculus vestigial like 1 homolog (Drosophila) (Vgll1) |
